# Thymic stromal lymphopoietin-stimulated CD4^+^ T cells induce senescence in advanced breast cancer

**DOI:** 10.3389/fcell.2022.1002692

**Published:** 2022-11-17

**Authors:** Margherita Boieri, Emanuela Marchese, Quan Minh Pham, Marjan Azin, Lauren E. Steidl, Anna Malishkevich, Shadmehr Demehri

**Affiliations:** ^1^ Center for Cancer Immunology, Center for Cancer Research, Massachusetts General Hospital and Harvard Medical School, Boston, MA, United States; ^2^ Cutaneous Biology Research Center, Department of Dermatology, Massachusetts General Hospital and Harvard Medical School, Boston, MA, United States

**Keywords:** TSLP, CD4^+^ T cell, breast cancer, cellular senescence, cancer immunotherapy, TNF-α (tumor necrosis factor-alpha), IFN-γ (interferon-gamma)

## Abstract

Thymic Stromal Lymphopoietin (TSLP) plays a prominent role in inducing type 2 immune response, commonly associated with atopic diseases. TSLP-activated CD4^+^ T helper 2 cells block early carcinogenesis by inducing terminal differentiation in spontaneous breast and lung cancer models. However, the impact of TSLP induction on advanced cancer with altered cellular phenotypes is unclear. Using an established MMTV-PyMt^tg^ breast cancer cell line, we demonstrate that TSLP-stimulated CD4^+^ T cells possess an antitumor effect in advanced breast cancer. In contrast to early breast cancer suppression, the antitumor immunity mediated by TSLP-stimulated CD4^+^ T cells in advanced breast cancer is mediated by the induction of a senescent-like phenotype in cancer cells. Inflammatory CD4^+^ T cells drive breast cancer cells into senescence by releasing interferon-gamma and tumor necrosis factor-alpha, which directly bind to their receptors on cancer cells. Our findings reveal a novel mechanism of TSLP-activated CD4^+^ T cell immunity against advanced breast cancer, mediated by cellular senescence as a distinct effector mechanism for cancer immunotherapy.

## Introduction

Recent advances in the field of tumor immunology have led to the development of immunotherapies that can harness the immune system to treat cancer. These strategies, such as immune checkpoint blockade and chimeric antigen receptor T cells (Pedroza-Gonzalez et al., 2011), mainly aim at reactivating tumor-infiltrating CD8^+^ cytotoxic T lymphocytes (CTLs) against late-stage metastatic cancers (Mellman et al., 2011). CD4^+^ T helper 2 (Th2) cells have been recently shown to possess the potential to efficiently eliminate early-stage tumor formation (Demehri et al., 2016; Liu et al., 2020). However, the potential efficacy of activating CD4^+^ T cells against late-stage cancers remains largely unexplored. Traditionally, CD4^+^ T cells are known to regulate the activity of other immune cells *via* the production of cytokines during inflammation. CD4^+^ T cells can differentiate into subsets with different effector functions. Th1, Th2, Th17, T follicular helper (Tfh), and regulatory T cells (Treg) are characterized by the expression of transcriptional marker T-bet, GATA3, RORγt, Bcl6, and FOXP3, respectively (Golubovskaya and Wu, 2016). Among the different subsets of CD4^+^ T cells, Th1 cells are known to play an essential role in the immune response against intracellular pathogens, such as bacteria and protozoa, by producing Th1 cytokines such as TNF-α and IFN-γ (Szabo et al., 2003; Zhu and Paul, 2008; Belizario et al., 2016). In contrast, Th2 cells play a significant role in the immune response against extracellular pathogens, such as helminths, by producing Th2 cytokines such as IL-3, IL-4, IL-5, and IL-13 (Constant and Bottomly, 1997; Bonecchi et al., 1998; Belizario et al., 2016).

Thymic Stromal Lymphopoietin (TSLP) and Th2 immunity have been extensively studies in the context of atopic diseases like asthma and atopic dermatitis (Al-Shami et al., 2005; Liu, 2006; Shikotra et al., 2012; Ziegler, 2012). TSLP and Th2 cells are found to play complex roles in cancer. On one hand, TSLP and Th2 immunity has been implicated in promoting tumor progression and metastasis. Studies have shown an association between the presence of Th2 cells in the tumor microenvironment (TME) and cancer progression in breast and lung cancers (DeNardo et al., 2009; Zaynagetdinov et al., 2015; Tokumaru et al., 2020). TSLP, at baseline level, has been shown to act directly on tumor cells and induce anti-apoptotic Bcl-2 expression (Kuan and Ziegler, 2018). Additionally, in response to TSLP, tumor cells have been shown to be secreting IL-1α that act on myeloid cells in the TME, which subsequently secrete TSLP back to tumor cells, creating an IL-1α/TSLP positive feedback loop (Olkhanud et al., 2011; Pedroza-Gonzalez et al., 2011; Kuan and Ziegler, 2018). On the other hand, TSLP induction is capable of driving inflammatory Th2 cell activation, which blocks carcinogenesis by inducing terminal differentiation in a spontaneous breast and lung cancer models (Demehri et al., 2016; Boieri et al., 2022; Guennoun et al., 2022). In addition, Th2 cell immunity plays a critical role in regulating tumor development, and depletion of Transforming Growth Factor (TGF)-β receptors on CD4^+^ T cells, but not CD8^+^ T cells, which can halt tumor progression in breast cancer (Liu et al., 2020). Importantly, most cancer-protective effects of TSLP/Th2 cells have been discovered in the context of early carcinogenesis using spontaneous cancer models.

We have previously shown that *K14-Tslp* ^*+/tg*^, *MMTV-PyMt* ^*+/tg*^ (Tslp^tg^, PyMt^tg^) mice are protected against early breast cancer development (Demehri et al., 2016; Boieri et al., 2022). However, it is unclear whether TSLP induction can also block tumor growth in advanced, metastatic breast cancer models. In this study, we investigated the role of TSLP induction during advanced breast cancer development using the PyMt cell line model in the context of TSLP induction in Tslp^tg^ mice (Demehri et al., 2016; Saxena et al., 2018). We found that classical Th1 cytokines, interferon-gamma (IFN-γ) and tumor necrosis factor-alpha (TNF-α), mediated the antitumor effect of TSLP-activated CD4^+^ T cells against advanced breast cancer through the induction of cellular senescence. Our findings provide novel insights into the mechanism of CD4^+^ T cell immunity and highlight TSLP induction as a potential therapeutic strategy in advanced breast cancer.

## Results

### Thymic stromal lymphopoietin induction protects against advanced breast cancer

**FIGURE 1:**
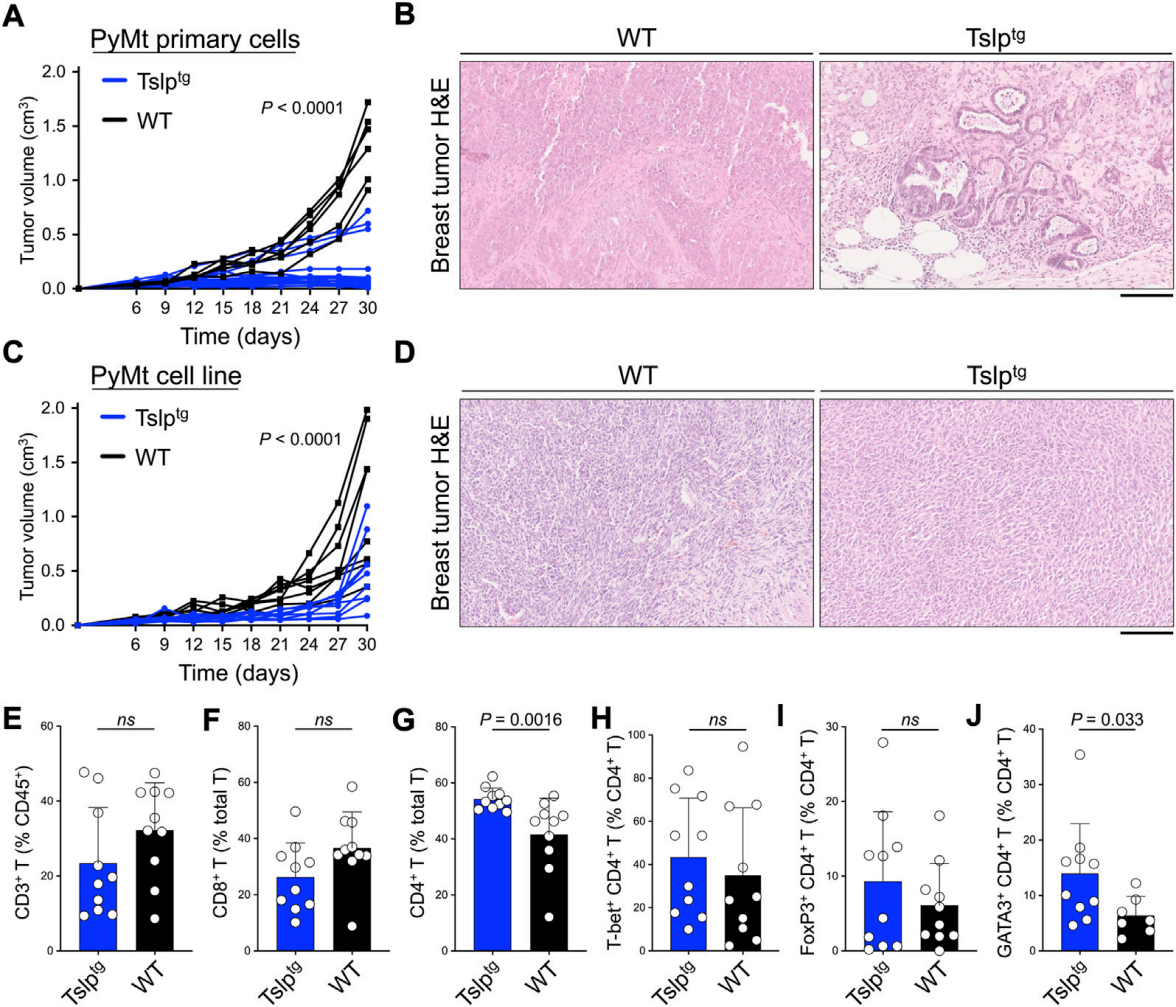
TSLP induction protects against early and advanced breast cancer. **(A)** PyMt^tg^ primary breast tumor growth in Tslp^tg^ (test, *n* = 19) versus WT (control, *n* = 6) mice (*p* < 0.0001, two-way ANOVA). **(B)** Representative images of hematoxylin and eosin (H&E)-stained PyMt primary breast tumors in Tslp^tg^ versus WT mice. **(C)** PyMt cell line-derived tumor growth in Tslp^tg^ (test, *n* = 9) versus WT (control, *n* = 8) mice (*p* < 0.0001, two-way ANOVA). **(D)** Representative images of H&E-stained PyMt cell line-derived tumors in Tslp^tg^ or WT mice. **(E–J)** Percent **(E)** CD3^+^ T cells among CD45^+^ cells, **(F)** CD8^+^ T cells among CD3^+^ T cells, **(G)** CD4^+^ T cells among CD3^+^ T cells, **(H)** T-bet^+^ Th1 cells among CD4^+^ T cells, **(I)** FoxP3^+^ Tregs among CD4^+^ T cells, and **(J)** GATA3^+^ Th2 cells among CD4^+^ T cells, collected from tumors and tumor-draining lymph nodes of Tslp^tg^ (test, *n* = 10) versus WT (control, *n* = 10) mice. *ns*: not significant, Mann-Whitney *U* test. Scale bars: 100 μm, bar graphs show mean + s.d.

To explore the antitumor effect of TSLP induction in early- versus late-stage breast cancer, we examined breast cancer development using the orthotopic breast tumor transfer model. Primary breast tumor cells from PyMt^tg^ mice or PyMt cell line were implanted into the abdominal mammary fat pad of Tslp^tg^ and wild-type (WT) controls on the C57BL/6 background. Tslp^tg^ mice that received primary tumor had delayed tumor growth and smaller tumors at endpoint compared with WT mice (*p* < 0.0001, Figure 1A). Transfer of primary tumors into Tslp^tg^ mice led to development of low-grade fibrocystic-like tumors compared with high-grade tumors in WT mice (Figure 1B) (Boieri et al., 2022). Tslp^tg^ mice receiving PyMt cell line also showed significantly delayed tumor growth and smaller tumors compared with WT mice (*p* < 0.0001, Figure 1C). However, histological analysis of the resected breast tumors from Tslp^tg^ and WT implanted with PyMt cell line did not show a change in cellular morphology between the two groups (Figure 1D). Furthermore, Tslp^tg^ mice PyMt cell line-derived breast tumor did not show a considerable number of apoptotic cells compared to WT mice suggesting that the suppressed breast tumor growth in Tslp^tg^ mice was not mediated by apoptosis induction (Supplementary Figure S1). Flow cytometry analysis of PyMt cell line-derived breast tumors and tumor-draining lymph nodes from Tslp^tg^ and WT mice revealed increased CD4^+^ T cells, especially GATA3^+^ CD4^+^ T cells in Tslp^tg^ compared with WT mice (Figures 1E–J). These findings demonstrate that TSLP induction protects against advanced breast tumor growth, which associates with the induction of CD4^+^ T cells against the cancer cells.

### CD4^+^ T cell supernatant alone is sufficient to suppress PyMt cell proliferation

**FIGURE 2:**
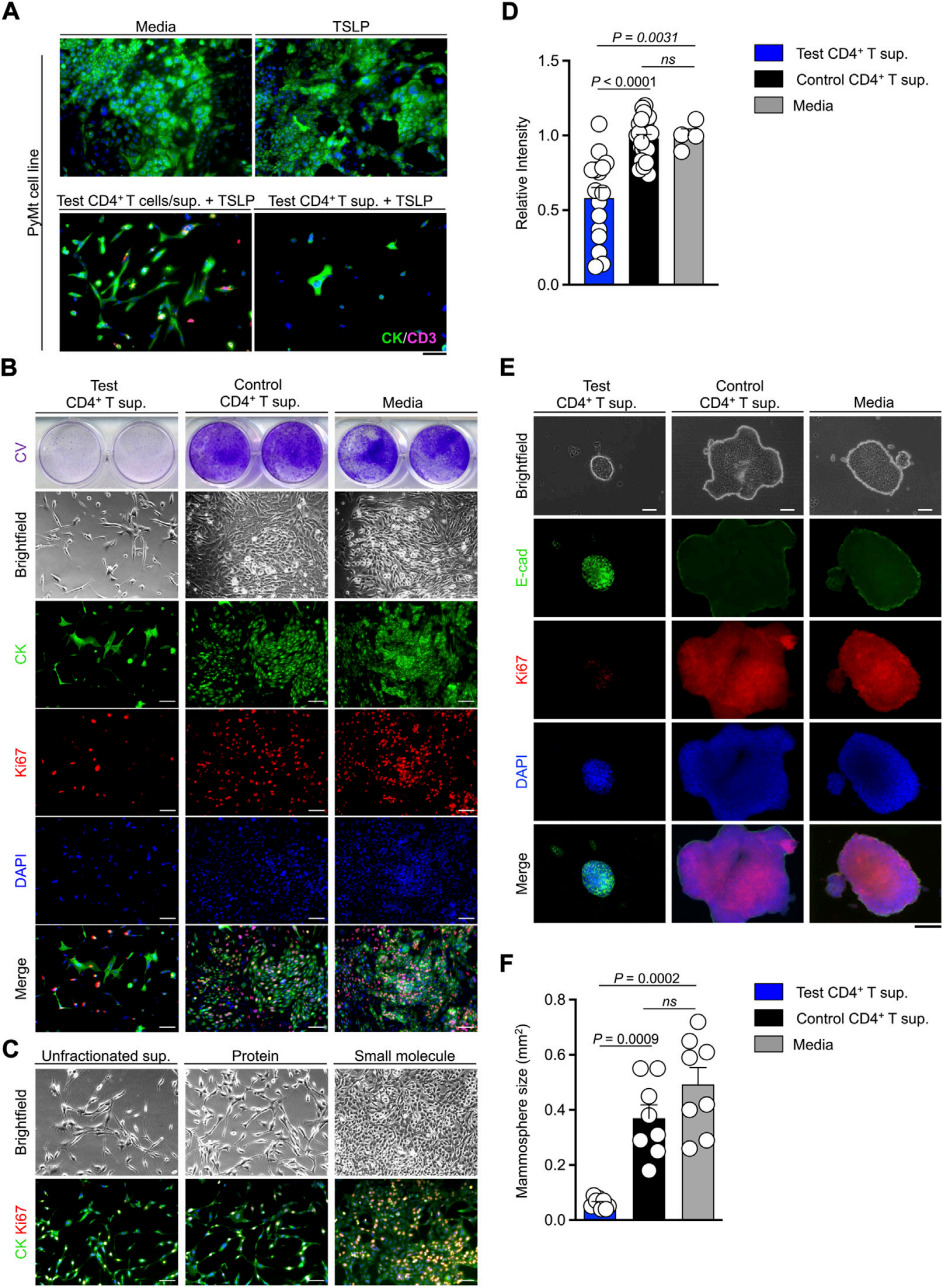
TSLP-activated CD4^+^ T cell supernatant blocks PyMt cell proliferation *in vitro*. **(A)** Representative images of cytokeratin (CK) and CD3^+^ T cells (CD3) immunofluorescence staining on 2D PyMt culture treated with media alone (top left), media plus TSLP (top right), TSLP-activated WT CD4^+^ T cells (test CD4^+^ T cells), their supernatant (sup.) and TSLP (bottom left), or CD4^+^ T cell supernatant and TSLP (bottom right). **(B)** Representative images of 2D proliferation assay with crystal violet staining (upper panels), brightfield (middle panels), and immunofluorescence staining for CK/Ki67/DAPI (lower panels) on PyMt cell line treated with test CD4^+^ T supernatant, control Tslpr^KO^ CD4^+^ T supernatant, or media alone. Supernatants were from the second and third rounds of CD4^+^ T cell stimulation *ex vivo*. **(C)** Representative images of 2D proliferation assay with brightfield (top panels) and immunofluorescence staining for CK/Ki67 (lower panels) on PyMt cell line treated with unfractionated test CD4^+^ T supernatant, protein fraction from test CD4^+^ T supernatant, or small molecule fraction from test CD4^+^ T supernatant. **(D)** Quantification of crystal violet-stained PyMt cells cultured in test CD4^+^ T supernatant (*n* = 16), control CD4^+^ T cell supernatant (*n* = 23) and control media (*n* = 4) conditions. Relative intensity of crystal violet stain in each well is determined using ImageJ. Each dot represents one well of culture plate (*ns*: not significant, Mann Whitney *U* test). **(E)** Representative images of 3D PyMt mammosphere brightfield (upper panels), immunofluorescence staining for E-cadherin(E-cad)/Ki67/DAPI (lower panels) after exposure to test CD4^+^ T supernatant, control CD4^+^ T supernatant, or media alone. **(F)** Quantification of mammosphere size in test CD4^+^ T cell supernatant (*n* = 8), control CD4^+^ T cell supernatant (*n* = 8) and control medium (*n* = 8). Each dot represents one mammosphere (*ns*: not significant, Mann Whitney *U* test). Scale bars: 100 μm, bar graphs show mean + s.d.

To determine whether TSLP-stimulated CD4^+^ T cells can directly affect the proliferation of PyMt cells, we developed an *in vitro* culture system. CD4^+^ T cells were sorted from tumors and tumor-draining lymph nodes of *Tslp* transgenic (test) or *Tslpr* knockout (Tslpr^KO^, control) mice and stimulated *ex vivo* using anti-CD3/CD28 antibodies plus TSLP. T cells supernatants were collected at the end of stimulation. The addition of TSLP to the culture media was critical to stimulate CD4^+^ T cells *ex vivo*. Because of the presence of TSLP in the culture media, we used sorted CD4^+^ T cells from Tslpr^KO^ mice as controls. PyMt cells were cultured in media alone, media supplemented with TSLP, TSLP-activated WT CD4^+^ T cells (test) and their supernatant, or test CD4^+^ T cell supernatant only. After 72 h in culture, immunofluorescence staining showed significantly lower cytokeratin signal reflecting lack of PyMt cell proliferation in culture with test CD4^+^ T cells or test CD4^+^ T supernatant alone compared with cells grown in media or media plus TSLP (Figure 2A). These results suggest that TSLP-stimulated CD4^+^ T cells secrete factor(s) that can block the growth of PyMt cells *in vitro*.

Next, we performed PyMt cell proliferation assay, where PyMt cells were treated with test CD4^+^ T supernatant, control CD4^+^ T supernatant, or media alone (Figure 2B). Crystal violet staining, brightfield, and immunofluorescence images of PyMt cells showed a significantly lower cell proliferation rate in PyMt cultured in test CD4^+^ T supernatant compared with control CD4^+^ T supernatant (*p* < 0.0001) and media (*p* = 0.0031) at 72 h after exposure to test and control CD4^+^ T supernatants (Figure 2D). To investigate which soluble agents produced by TSLP-stimulated CD4^+^ T cells could be responsible for blocking PyMt cell proliferation, we fractionated the test CD4^+^ T cell supernatant based of the molecular mass of 3 kilodalton (kDa) and cultured PyMt cells in the different fractions. Both brightfield and CK/Ki67 immunofluorescence imaging demonstrated that while the small molecule fraction (<3 kDa) of the test CD4^+^ T supernatant did not show any growth suppressing effect, both unfractionated and the protein fraction (>3 kDa) fraction of the test CD4^+^ T supernatant blocked PyMt cell proliferation (Figure 2C). These results suggest that proteins including cytokines secreted by TSLP-activated CD4^+^ T cells are likely responsible for the suppression of PyMt cell proliferation.

PyMt cell line was exposed to the supernatant from the CD4^+^ T cells in a 3D mammosphere culture system and stained for E-cadherin as a marker of epithelial cells and Ki67 to mark proliferating cells. At 7 days after treatment with T cell supernatants, PyMt cells cultured in the supernatant from test CD4^+^ T cells formed small spherical mammospheres with low proliferation, while PyMt cells cultured in control CD4^+^ T cell supernatant formed large and irregularly shaped mammospheres with a higher number of Ki67^+^ cells (Figures 2E,F). Collectively these results show that TSLP-stimulated CD4^+^ T cells produce soluble factors that block the proliferation of PyMt cells *in vitro*.

### CD4^+^ T cell supernatant causes PyMt cell senescence

**FIGURE 3:**
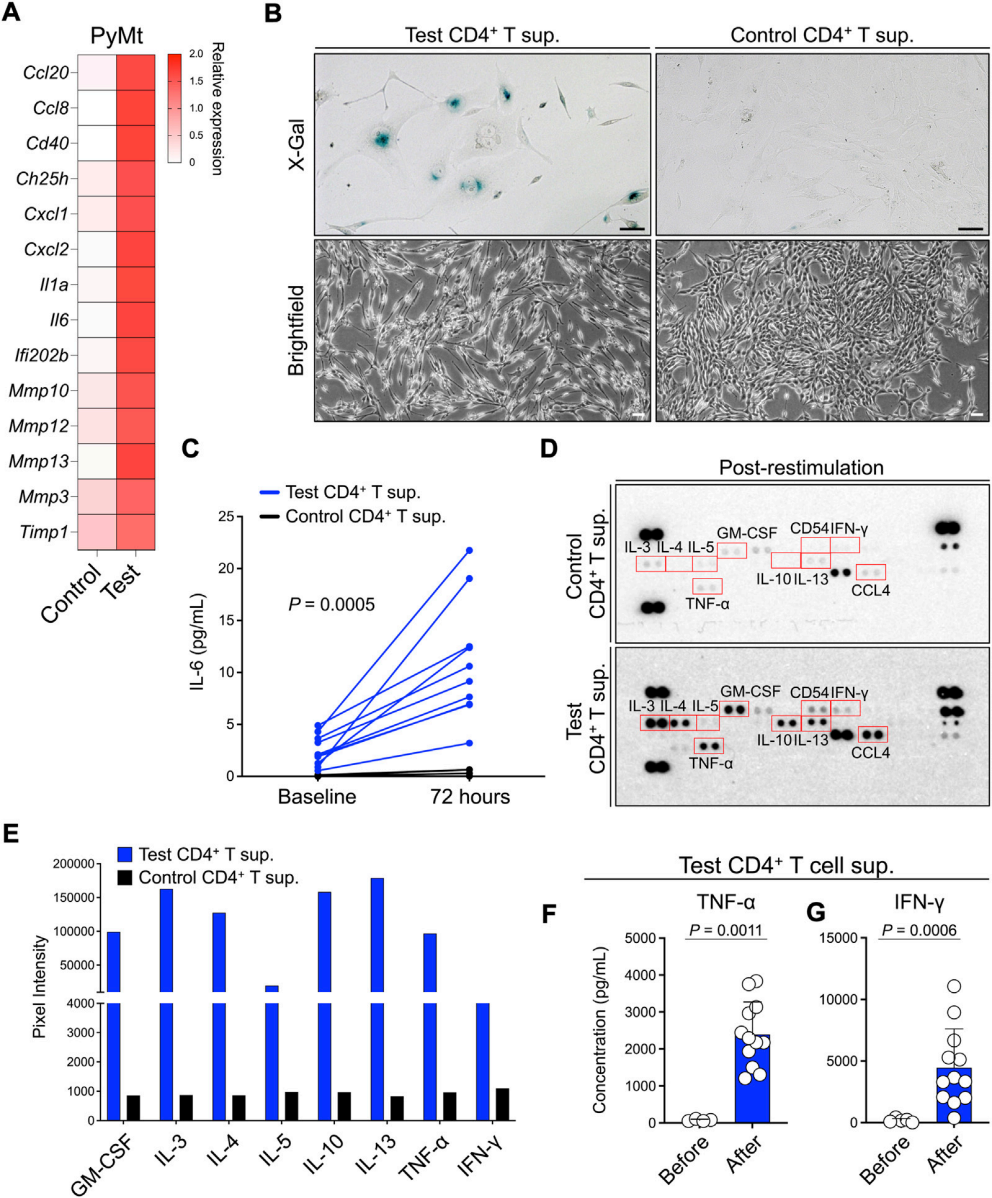
TSLP-activated CD4^+^ T cell supernatant induces senescence-like phenotype in PyMt cells. **(A)** The transcriptome analysis for SASP gene expression in PyMt cells treated with TSLP-activated WT CD4^+^ T cell supernatant (sup.) (test, *n* = 6) versus Tslpr^KO^ CD4^+^ T supernatant (control, *n* = 4). The heatmap demonstrates the ratio between mean fragments per kilobase of transcript per million mapped reads (FPKM) value of each group and the average of mean FPKM values of the two groups. **(B)** Representative images of X-Gal staining (upper panels) and brightfield (lower panels) of PyMt cells treated with test versus control CD4^+^ T supernatant (scale bars: 100 μm). The beta-galactosidase activity was detectable at pH 6.0. **(C)** Quantification of IL-6 concentration, measured by ELISA, in test (*n* = 10) versus control CD4^+^ T cell supernatants (*n* = 8) before and 72 h after in PyMt cell culture (*p* = 0.0005, two-way ANOVA). **(D)** Representative mages of protein arrays performed on CD4^+^ T cells supernatants from the control (top panel) and test T cells (bottom panel). Supernatants used in this assay were collected after CD4^+^ T cell stimulation *in vitro*. Red boxes highlight upregulated cytokines in test compared with control CD4^+^ T cells supernatant, including Th1-associated cytokines (TNF-α and IFN-γ) and Th2-associated cytokines (IL-3, IL-4, IL-5, IL-13, GM-CSF). **(E)** Quantification of cytokines upregulated in test CD4^+^ T cell supernatant on protein arrays using ImageJ. **(F,G)** Quantification of **(F)** TNF-α and **(G)** IFN-γ concentrations, measured by ELISA, in test CD4^+^ T cell supernatants before (*n* = 4) and after restimulation (*n* = 12), Mann-Whitney *U* test. Post-restimulation supernatants were collected after three cycles of stimulation with anti-CD3/CD28 antibodies plus TSLP. Bar graphs show mean + s.d. The same amount of total protein for each sample was used for protein detection and quantification in **(D–G)**.

To determine the mechanism involved in the arrest of PyMt proliferation when exposed to test CD4^+^ T cell supernatant, we performed RNA sequencing on the PyMt cells after culture with test versus control CD4^+^ T cells supernatants. Among the top 20 differentially upregulated genes in the cells that were exposed to test CD4^+^ T supernatant, several genes, such as interleukin (IL)-6, matrix metallopeptidase 13, and interleukin 1 alpha, belonged to the senescence-associated secretory phenotype (SASP, Figure 3A) (Coppe et al., 2010a; Coppe et al., 2010b; Hudgins et al., 2018). To examine whether PyMt cells exposed to test CD4^+^ T supernatant undergo senescence, we performed a beta-galactosidase assay (X-gal staining) on test versus control CD4^+^ T supernatant-treated PyMt cells. PyMt cells cultured in test T supernatant showed X-gal positive signals while control cells had no X-gal signal (Figure 3B). In addition, IL-6 was secreted at higher levels by PyMt cells cultured in test compared with control CD4^+^ T supernatant (Figure 3C). Consistent with these results, PyMt cell line-derived breast tumors from Tslp^tg^ mice showed the activation of p21/p53 axis compared with WT mice supporting the induction of senescence by TSLP-activated CD4^+^ T cells *in vivo* (Supplementary Figure S2).

To determine the factor(s) released by TSLP-stimulated CD4^+^ T cells that are responsible for inducing PyMt senescence, we analyzed components of CD4^+^ T supernatants using a cytokines and chemokines array. In addition to the production of canonical Th2 cytokines such as IL-3, IL-4, IL-5 and IL-13, test CD4^+^ T supernatant contained higher levels of TNF-α and IFN-γ compared with control CD4^+^ T supernatant (Figures 3D,E). Consistent with the protein array results, after three rounds of stimulation with anti-CD3/CD28 antibodies plus TSLP, test CD4^+^ T cell supernatant showed significant upregulation in TNF-α (*p* = 0.0011) and IFN-γ (*p* = 0.0006) (Figures 3F,G). These findings demonstrate that Th1-associated cytokines, IFN-γ and TNF-α, are secreted by TSLP-stimulated inflammatory Th2 cells and suggest that these Th1-like cytokines may play a role in inducing senescence in advanced breast tumors.

### TNF-α and IFN-γ are required for thymic stromal lymphopoietin-activated CD4^+^ T cells effect against PyMt cells

**FIGURE 4:**
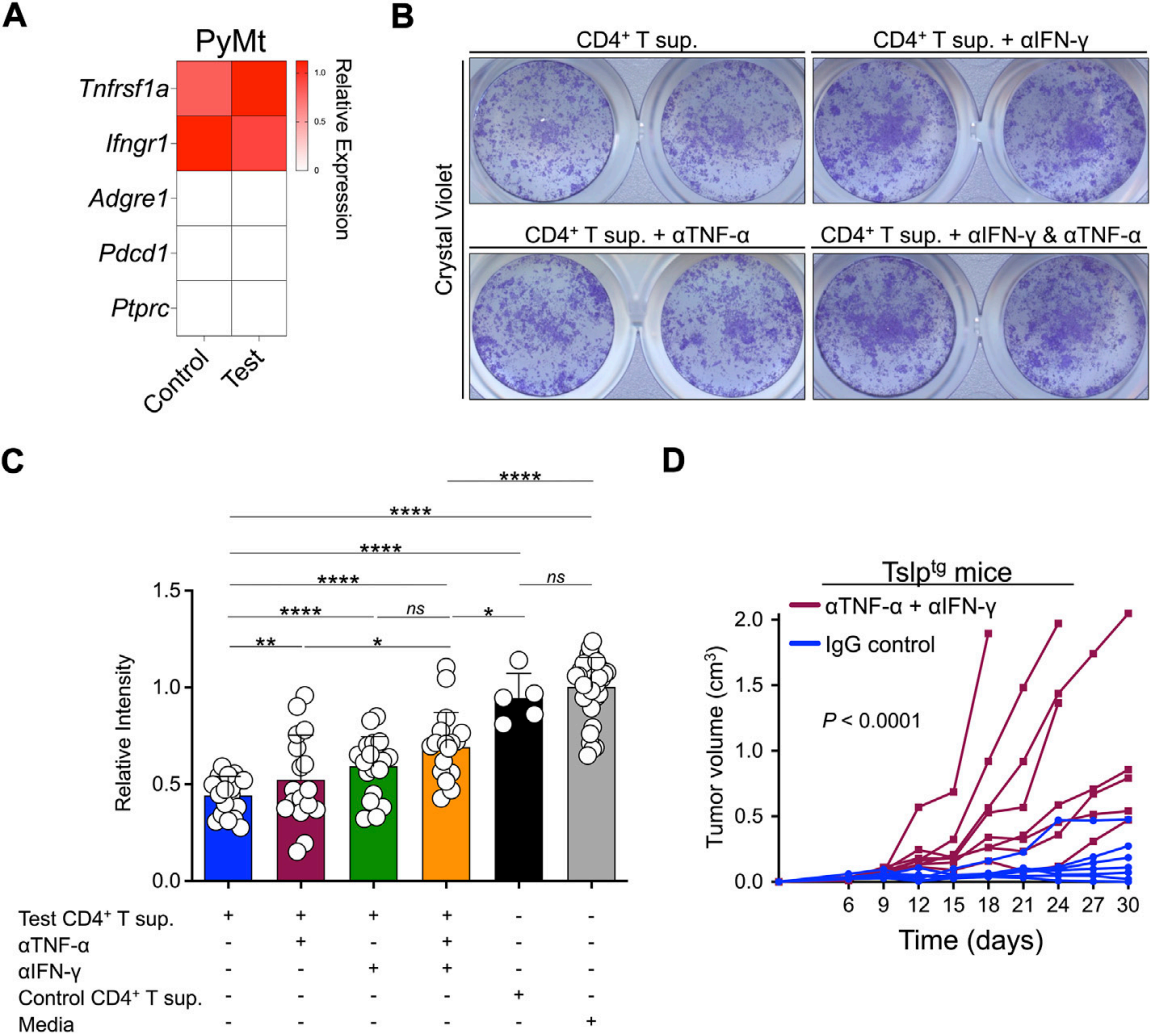
TNF-α and IFN-γ contribute to TSLP-activated antitumor immunity against advanced breast cancer. **(A)** The transcriptome analysis for TNF-α and IFN-γ receptors expression in PyMt cells treated with TSLP-activated WT CD4^+^ T cell supernatant (sup.) (test, *n* = 6) and Tslpr^KO^ CD4^+^ T supernatant (control, *n* = 4). *Adgre1*, *Pdcd1* and *Ptprc* are immune cell-related genes that are shown as negative controls. The heatmap demonstrates the ratio between mean fragments per kilobase of transcript per million mapped reads (FPKM) value of each group and the average of mean FPKM values of the two groups. **(B)** Representative images of 2D proliferation assay with crystal violet staining of PyMt cell line culture with test CD4^+^ T supernatant (top left), test CD4^+^ T supernatant + anti-IFN-γ blocking antibody (top right), test CD4^+^ T supernatant + anti-TNF-α blocking antibody (bottom left), and test CD4^+^ T supernatant + anti-IFN-γ and anti-TNF-α combined (bottom right). **(C)** Quantification of crystal violet-stained PyMt cells cultured in test CD4^+^ T cell supernatant (*n* = 10), test CD4^+^ T supernatant + anti-IFN-γ blocking antibody (*n* = 19), test CD4^+^ T Sup + anti- TNF-α blocking antibody (*n* = 17), test CD4^+^ T Sup + anti- TNF-α and anti-IFN-γ combined (*n* = 18), control CD4^+^ T supernatant (*n* = 5) and media alone (*n* = 36). Relative intensity of crystal violet stain in each well is determined using ImageJ (*: *p* < 0.05, **: *p* < 0.01, ***: *p* < 0.001, ****: *p* < 0.0001, *ns*: not significant, Mann-Whitney *U* test). Bar graphs show mean + s.d. **(D)** PyMt cell line-derived tumor growth in Tslp^tg^ mice treated with anti-TNF-α and anti-IFN-γ blocking antibodies (test, *n* = 8) versus IgG control (control, *n* = 7, *p* < 0.0001, two-way ANOVA).

To further investigate the contribution of TNF-α and IFN-γ to CD4^+^ T cell-induced PyMt cellular senescence, we confirmed the expression of their receptors (*Tnfrsf1* and *Ifngr1*, respectively) in PyMt cells (Figure 4A). Importantly, blocking TNF-α and IFN-γ led to a higher rate of proliferation in PyMt cells treated with test CD4^+^ T supernatant, with the greatest effect seen when blocking both cytokines (Figures 4B,C). In contrast, blocking Th2 cytokines did not affect the inhibition of PyMt cell proliferation by test CD4^+^ T cell supernatant (Supplementary Figure S3). To examine the role of TNF-α and IFN-γ in TSLP-induced PyMt tumor suppression *in vivo*, we implanted PyMt cells in Tslp^tg^ mice and blocked TNF-α and IFN-γ using blocking antibodies. The combination of anti-TNF-α and anti-IFN-γ blocking antibodies reversed the tumor-suppressing effect of TSLP induction and resulted in an accelerated breast tumor growth compared with IgG-treated group (*p* < 0.0001, Figure 4D). Although to lesser degree, TNF-α and IFN-γ blockade also accelerated tumor growth in WT mice (Supplementary Figure S4). Together, these findings indicate that TNF-α and IFN-γ produced by TSLP-stimulated inflammatory Th2 cells play a key role in providing antitumor immunity against advanced breast cancer.

## Discussion

The majority of current cancer immunotherapy strategies have focused on using CD8^+^ CTLs to treat advanced cancers (Mellman et al., 2011). However, the potential efficacy of inducing CD4^+^ T cell response to prevent and treat cancer remains mostly unexplored. Our findings demonstrate a role for TSLP induction in the treatment of advanced breast cancer. These data complement our previous observation that TSLP induction mounts a robust Th2 cell response preventing breast tumor development by inducing terminal differentiation of the early-stage breast cancer cells (Demehri et al., 2016; Boieri et al., 2022). Other studies have also shown that the Th2 cells can provide an antitumor effect during tumorigenesis (Nishimura et al., 1999; Mattes et al., 2003; Lorvik et al., 2016). Together, these findings provide evidence that TSLP induction to drive CD4^+^ T cell activation can potentially be an effective therapeutic strategy to prevent cancer development and progression in high-risk patients.

TSLP has been described to have a pro-tumor effect and can drive tumorigenesis both directly and indirectly. Previous studies have shown that TSLP act directly on tumor cells and provide anti-apoptotic signals sufficient to drive tumor growth (DeNardo et al., 2009; Zaynagetdinov et al., 2015; Tokumaru et al., 2020). Other studies have shown that TSLP can act to facilitate cancer development by suppressing the immune response. These studies have shown that the pro-tumor effect of TSLP is mediated by myeloid suppressor cells that can dampen the immune response, induce wound healing, and provide survival signals for tumor cells (Olkhanud et al., 2011; Kuan and Ziegler, 2018). However, these studies have investigated the baseline levels of TSLP expressed in TME. Thus, TSLP appears to play a dual role, providing both pro and antitumor immunity depending on its expression level and the tumor context.

Our findings demonstrate that TSLP-stimulated CD4^+^ T cell immunity can block breast cancer growth by inducing a cellular senescent phenotype in advanced breast tumors. We show that TSLP-stimulated CD4^+^ T cells are sufficient to establish this antitumor phenotype, which is associated with Th2 polarization. Instead of cellular cytotoxicity, the tumor-suppressive phenotype is mediated by cellular senescence, in which effector CD4^+^ T cells can directly block advanced breast tumor development. Remarkedly, this effector mechanism is mediated by inflammatory Th2 cells releasing Th1 cytokines IFN-γ and TNF-α, which bind to receptors on breast tumor cells. Interestingly, TSLP induction have previously been shown to activate inflammatory Th2 cell phenotype with high TNF-α expression (Ito et al., 2005). In addition, the canonical Th1 cytokines TNF-α and IFN-γ have also been shown to be able to induce cellular senescence in cancers as an alternative mechanism for arresting cancer progression (Braumüller et al., 2013; Rosemblit et al., 2018). Collectively, these results showcase the antitumor function of TSLP and CD4^+^ T cells, which can be a promising therapeutic strategy for cancer immunoprevention and treatment.

The discovery of inflammatory Th2 cells as direct mediators of antitumor immunity in advanced breast carcinogenesis provides fundamental insights into the plasticity of CD4^+^ T cell lineage during immune response. In addition to secreting the canonical Th2 cytokines, IL-4, IL-5, and IL-13, TSLP-stimulated GATA3^+^ CD4^+^ T cells are able to secrete TNF-α and IFN-γ. Thus, a novel antitumor mechanism driven by TSLP-stimulated CD4^+^ T cells and delivered by TNF-α and IFN-γ cytokines results in the senescence of advanced breast cancer cells. Collectively, our research on TSLP induction in breast cancer reveals that during the early stages of breast cancer development, cancer cells respond to TSLP-activated CD4^+^ T cell immunity by undergoing terminal differentiation (Boieri et al., 2022). In contrast, advanced cancer cells are no longer capable of initiating a differentiation program and they respond to TSLP-activated CD4^+^ T cell immunity by undergoing senescence. As such, terminal differentiation and senescence represent novel cancer-suppressing immune mechanisms in the early and late stages of breast cancer, respectively.

### Limitations of this study

We did not have access to CD4-specific Tslpr knockout mice to study whether TSLP-mediated antitumor immunity is mediated solely by CD4^+^ T cells. Although CD8^+^ T cells did not increase in the Tslp^tg^ tumors, it is possible that CD8^+^ T and natural killer cells contributed to the observed tumor suppression phenotype in response to TSLP induction as TSLP receptor is broadly expressed on immune cells. Further, we have only used Tslp^tg^ transgenic mice as our model of TSLP induction. Future studies are warranted to determine the effect of transient TSLP induction on advanced breast cancer.

## Materials and methods

### Study approval

Animal studies were approved by Massachusetts General Hospital Institutional Animal Care and Use Committee (IACUC).

### Mice

All mice were housed under specific pathogen-free conditions with a 12-h light-dark cycle and given water and food *ad libitum* in the animal facility at the Massachusetts General Hospital (MGH) in compliance with animal care and all other relevant regulations. *MMTV-PyMt* ^*+/tg*^ (PyMt^tg^, provided by Dr. David DeNardo, Washington University in St. Louis, St. Louis, MO, United States), *K14-Tslp* ^*+/tg*^ (Tslp^tg^, provided by Dr. Andrew Farr, University of Washington, Seattle, WA, United States), and *Tslpr* ^*−/−*^ (Tslpr^KO^, provided by Dr. Warren Leonard, National Institute of Health, Bethesda, MD, United States). All mice in this study were maintained on the C57BL/6 backgrounds. Age-matched female mice were used in all the breast cancer studies.

### CD4^+^ T cell sorting and supernatant collection

Tumor and tumor-draining lymph nodes collected from Tslp^tg^ (test) and Tslpr^KO^ (control) mice were processed into a single cell suspension and stained with anti-CD3ε, anti-CD4, anti-CD8α, anti-CD19 and anti-NK1.1 antibodies. CD4^+^ T cells were sorted on a BD FacsARIA (BD Biosciences, San Jose, CA, United States) or SONY SH800 sorter (Sony Biotechnologies, San Jose, CA, United States) and plated at the concentration of 0.5 × 10^6^ cells/m in anti-CD3 coated plates in RPMI medium 1,640 (Gibco, Carlsbad, CA, United States), supplemented with 10% fetal bovine serum (Corning, Manassas, VA, United States), 1% penicillin/streptavidin/glutamine (Thermo Fisher Scientific), 22 μM 2-mercaptoethanol (Gibco), 50 U/ml IL-2 (BioLegend, San Diego, CA, United States), 20 ng/ml TSLP (Thermo Fisher Scientific) and 2 μg/ml anti-CD28. 72 h later, supernatants were harvested, filtered through a 22 μm filter, and stored at −20°C until use. CD4^+^ T cells were plated again at 0.5 × 10^6^ cells/ml in 12-wells plates in the same medium without anti-CD3/CD28 and cultured for 48 h. The stimulation-resting cycle was repeated for a total of three stimulations, and supernatants were collected at the end of each stimulation. T cell supernatant fractionation was performed using 3 kDa Amicon^®^ Pro Purification System with Ultra-0.5 Device following manufacturer instructions (catalog no. ACS500312, MilliporeSigma, Burlington, MA, United States).

### PyMt cell transfer

PyMt cell line was derived from PyMt^tg^ tumors and expanded *in vitro* in D10 media consisting of DMEM (Gibco, Carlsbad, CA, United States) supplemented with 10% fetal bovine serum (Corning, Manassas, VA, United States), 1% penicillin/streptavidin/glutamine (Thermo Fisher Scientific), 22 μM 2-mercaptoethanol (Gibco) (Demehri et al., 2016). Once reached 60%–80% confluence, PyMt cells were washed and resuspended in PBS and mixed with Matrigel™ (Corning), with a 1:1 ratio. PyMt cells in a mixture of PBS: Matrigel™ were injected into the left mammary fat pad of the animals.

### Flow cytometry

Breast tumors and tumor-draining lymph nodes were incubated with collagenase IV (Worthington) for 2 h at room temperature and filtered through a 70 μm cell strainer to obtain a single-cell suspension. Tumor-infiltrating leucocytes were isolated by immunomagnetic separation with CD45 MicroBeads and magnetic columns (Miltenyi Biotec, Bergisch Gladbach, Germany). Lymph node and tumor cells were stained with the following monoclonal surface antibodies: anti-CD3ε, anti-CD4, anti-CD8α, anti-CD45 and anti-CD19. Next, cells were fixed and permeabilized by True-Nuclear Transcription Factor Buffer Set (Biolegend). Intracellular staining was performed using the following antibodies: anti-T-bet, anti-GATA3, anti-Foxp3, and anti-Ki67 (Biolegend, San Diego, CA, United States). Stained cells were assayed using a Fortessa LSRII flow cytometer (BD Bioscience), and data were analyzed using FlowJo software (Tree Star, Ashland, OR).

### Histology, immunofluorescence, and immunohistochemistry

Tissue samples were harvested and fixed in 4% paraformaldehyde (Sigma-Aldrich) solution in PBS overnight at 4°C. Samples were processed and embedded in paraffin. Paraffin-embedded tissues were cut at 5 μm and stained for Hematoxylin and Eosin (H&E). For immunofluorescence (Guennoun et al., 2022) staining, sections were incubated with CD3 (catalog no. Ab11089, Abcam, Waltham, MA), cytokeratin [catalog no. M3515, Dako (Agilent), Santa Clara, CA, United States] and Ki67 (Catalog no. Ab15580, Abcam) antibodies followed by fluorochrome-conjugated secondary antibodies. Nuclei were counterstained with DAPI (Thermo Fisher Scientific). Slides were scanned using the NanoZoomer s60 digital scanner (Hamamatsu Corp. Bridgewater, NJ United States), and high-resolution images were acquired using a Zeiss Axio Observer Z1 (Zeiss, Oberkochen, Germany). Images processing and analysis was performed using ZEN Image Processing software (Zeiss). Crystal violet (catalog no. C6158, Sigma-Aldrich, St. Louis, MO, United States), beta-galactosidase assay (catalog no. 11828673001, Sigma-Aldrich) and TUNEL assay (catalog no. 48513, Cell Signaling Technology) were conducted following the manufacturer’s instructions. For TUNEL assay, paraffin embedded samples were cut at 5 μm and processed for deparaffinization and rehydration. Samples were subjected to antigen retrieval unmasking solution for 10 min using pressure cooker. After incubation with TUNEL reaction buffer, samples were incubated with TdT Enzyme (Terminal deoxynucleotidyl Transferase) for 1 h at 37°C protected from the light for the labeling reaction. Sections were counterstained with DAPI nuclear stain and high-resolution images were acquired using a Zeiss Axio Observer Z1 (Zeiss) and analyzed using the ZEN Image Processing software.

### RNA and protein isolation

For RNA extraction, breast tumor samples were homogenized in RLT buffer (Qiagen, Valencia, CA, United States) supplemented with 1% 2-mercaptoethanol (Thermo Fisher Scientific) using a TissueLyser II (Qiagen) at a frequency of 30/s for 5 min. Homogenized tissue were resuspended in TRIzol™ Reagent (Thermo Fisher Scientific) and stored at −80°C until use. Sorted CD4^+^ T cells for RNA sequencing were resuspended in RLT buffer (Qiagen) supplemented with 1% 2-mercaptoethanol (Thermo Fisher Scientific) and stored at −80°C until use. RNA was isolated using the RNeasy Mini Kit (Qiagen), quantified using a NanoDrop ND-1000 spectrophotometer, and stored at −80°C until use.

For protein extraction, breast tumor samples were homogenized in phosphate-buffered saline (PBS) supplemented with 0.1% v/v Tween 20 (Sigma-Aldrich) and 4% protease inhibitor (Thermo Fisher Scientific) using a TissueLyser II (Qiagen) at a frequency of 30/s for 5 min. Samples were transferred to new tubes, frozen in liquid nitrogen for 1 min, and then thawed in a 37°C water bath for 3 min. Subsequently, samples were sonicated for 1 min, followed by centrifugation at 13,300 rpm for 10 min at 4°C. The aqueous phases containing protein extract was transferred to a new tube and stored at −80°C until use.

### Mammosphere culture

The mouse PyMt cell line was used for 3D *in vitro* experiments. PyMt cells were plated in low adherence 48-wells plate in RPMI medium 1,640 (Gibco), supplemented with 10% fetal bovine serum (Corning), 1% penicillin/streptavidin/glutamine (Thermo Fisher Scientific), 22 μM 2-mercaptoethanol (Gibco), and 5 ug/ml insulin (medium) or in growth media containing test versus control CD4^+^ T cell supernatants. Pierce BCA Protein Assay Kit (Thermo Fisher Scientific) was used to measure the total protein concentration in the supernatants to have the same amount of protein in each assay. Seven days after the addition of the T cell supernatants, mammospheres were counted under an inverted fluorescence microscope, and pictures of all mammospheres were taken. The size of mammospheres was measured using ImageJ. For immunofluorescence staining, mammospheres were collected and centrifuged onto slides using a Cytospin 4 Cytocentrifuge (Thermo Fisher Scientific) at 300 rpm for 3 min. Samples were fixed in methanol at −20°C for 20 min. Slides were permeabilized in PBS supplemented with 0.3% v/v Triton X-100 (Thermo Fisher Scientific) for 30 min and blocked with PBS supplemented with 0.1% v/v Tween 20 (Sigma-Aldrich), 5% (m/v) bovine serum albumin (Thermo Fisher Scientific) and 10% (v/v) goat serum (Sigma-Aldrich) for 1 h. Mammospheres were stained with primary antibodies mouse anti-E-Cadherin (catalog no. 610181, BD Biosciences, Woburn, MA) and Ki67 at 4°C overnight. Subsequently, samples were incubated with secondary antibodies for 2 h at room temperature protected from the light. For nuclear staining, slides were incubated with DAPI (Invitrogen) for 10 min and mounted with Prolong Gold Antifade Reagent (Invitrogen). Pictures were acquired on a Zeiss Axio Observer Z1 (Zeiss, Oberkochen, Germany) and analyzed using the Zeiss ZEN Image Processing software.

### Protein array

CD4^+^ T cell culture supernatants were thawed, and total protein concentrations were measured with a Pierce BCA Protein Assay Kit (Thermo Fisher Scientific) according to manufacturers’ instructions. Optical densities were measured on a Synergy Neo2 (Biotek) at 562 nm. Measurements were normalized, and total protein concentrations were calculated with a four-parameter logistic curve using Gen5 Microplate Reader and Imager Software (Biotek). Equal amounts of total protein in CD4^+^ T cell culture supernatants were used to screen and quantify cytokine production using the Proteome Profiler Mouse XL Cytokine Array (R&D Systems, MN, Canada) following manufacturer’s instructions. Spot intensity was quantified using Gilles Carpentier’s Dot-Blot-Analyzer macro (written by Gilles Carpentier, 2008. Available at http://rsb.info.nih.gov/ij/macros/toolsets/Dot%20Blot%20Analyzer.txt, more information can be found at http://image.bio.methods.free.fr/dotblot.html) written for ImageJ (version 2.0.0).

### Enzyme-linked immunosorbent assay

Expression of TNF-α and IFN-γ cytokines was measured on CD4^+^ T cell supernatants using enzyme-linked immunosorbent assay (ELISA), LEGEND MAX™ Mouse TNF-α ELISA Kit and LEGEND MAX™ Mouse IFN-γ ELISA Kits (BioLegend), and following the manufacturer’s instructions. IL-6 expression was measured from PyMt culture media after treatment using the LEGEND MAX™ Mouse IL-6 ELISA Kit (BioLegend). The same amount of total protein, quantified using Pierce BCA Protein Assay Kit (Thermo Fisher Scientific), was used for the assays. Optical densities were measured on a Synergy Neo2 (Biotek, Winooski, VT, United States) at 450 nm and cytokine concentrations were calculated with a five-parameter logistic curve using Gen5 Microplate Reader and Imager Software (Biotek).

### Genotyping

PCR was used for genotyping genetically engineered mice. Primer pairs used in this study are described in Supplementary Table S1. All primers shown are 5′ to 3′.

### RNA sequencing

PyMt cells after culture with test versus control CD4^+^ T cells supernatants were collected and the total RNA were sent to Beijing Genomics Institute (BGI) for RNA sequencing. Libraries were prepared by BGI, quantified and qualified using the Agilent 2100 Bioanalyzer and ABI StepOnePlus Real-Time PCR System and sequenced using Illumina HiSeqTM 2000. RNA sequencing data were analyzed using the pipeline Version 5.0. Sequences were aligned to the mouse reference genome (mm10) using Bowtie, and differentially expressed genes were screened using the Poisson distribution method, the Noiseq, or EBSeq packages. Original data are available at the NCBI Gene Expression Omnibus (GEO), accession numbers: GSE214000.

### Statistical analysis

Graphs and statistical analysis were performed using GraphPad Prism 9 and RStudio. Bar graphs show mean + standard deviation (s.d.). Two-way ANOVA with Sidak’s multiple comparison test was used to compare tumor growth and IL-6 production over time between different groups. Two-tailed Mann-Whitney *U* test was used for all the other comparisons. A *p* value of less than 0.05 was considered significant. A *p* value of <0.05 was considered significant. All error bars represent standard deviation (s.d.).

## Data Availability

The datasets presented in this study can be found in online repositories. The names of the repository/repositories and accession number(s) can be found below: https://www.ncbi.nlm.nih.gov/geo/, GSE214000.
