# Supplementary material for: Thymic stromal lymphopoietin-stimulated CD4+ T cells induce senescence in advanced breast cancer
Source: Front Cell Dev Biol. 2022 Nov 17;10:1002692. doi: 10.3389/fcell.2022.1002692 (PMC9714463; doi:10.3389/fcell.2022.1002692)
Supplement: Supplementary file 1 [file DataSheet1.pdf]

Supplementary Material

Title: TSLP-Stimulated CD4$^+$ T Cells Induce Senescence in Advanced Breast Cancer

Authors: Margherita Boieri[1,2‡], Emanuela Marchese[1,2‡], Quan Minh Pham[1,2], Marjan Azin[1,2], Lauren E. Steidl[1,2], Anna Malishkevich[1,2], and Shadmehr Demehri[1,2*]

Supplementary Figures

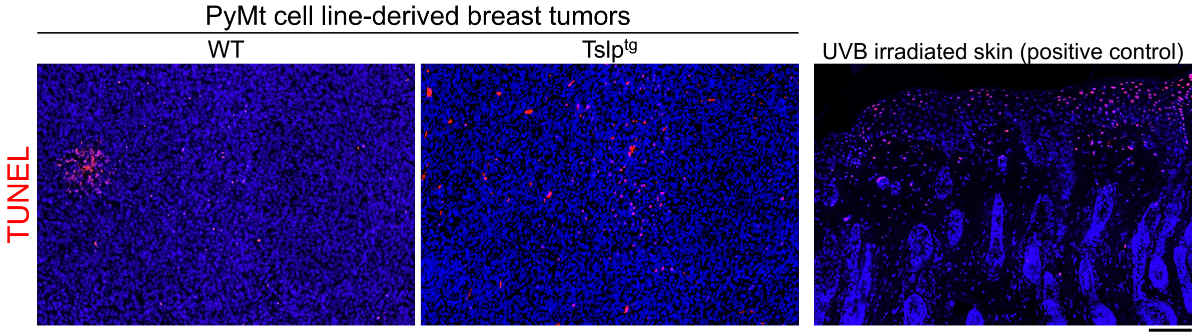

Figure S1. **TSLP induction protects against advanced breast cancer in an apoptosis-independent manner.**

Representative images of TUNEL assay on PyMt cell line-derived breast tumors in Tslp^{tg} and WT mice. TUNEL-stained dorsal skin from a Xpc$^{-/-}$ mouse that is irradiated with 100 mJ/cm2 ultraviolet B (UVB) is shown as a positive control. Nuclei are stained with DAPI in blue. Scale bar: 100 μm.

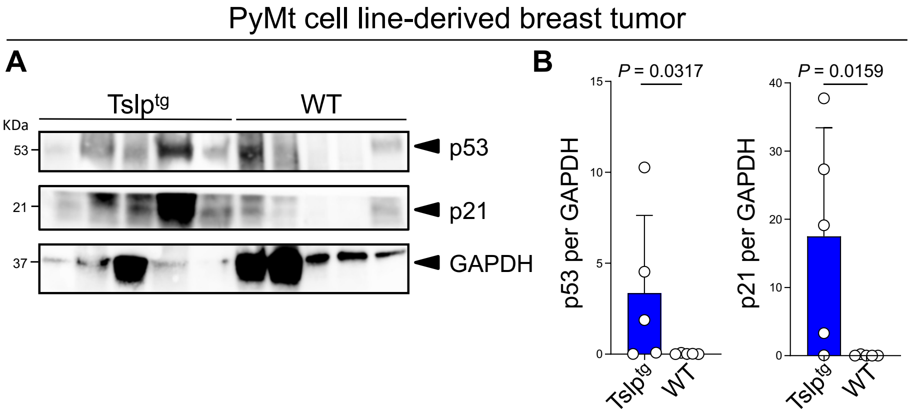

Figure S2. **TSLP induction protects against advanced breast cancer growth by inducing senescence in cancer cells.**

(**A**) Western blot for senescence markers (p21 and p53) on PyMt cell line-derived breast tumor lysates from $Tslp^{tg}$ and WT mice. GAPDH is used as the control housekeeping protein. Please note that the low levels of GAPDH in a few samples is due the very small size of tumors that developed in $Tslp^{tg}$ mice. (**B**) Quantification of p21 and p53 protein bands in western blot of PyMt cell line-derived breast tumors from $Tslp^{tg}$ (n=5) and WT (n=5) mice. Mann Whitney *U* test, bar graphs show mean + s.d.

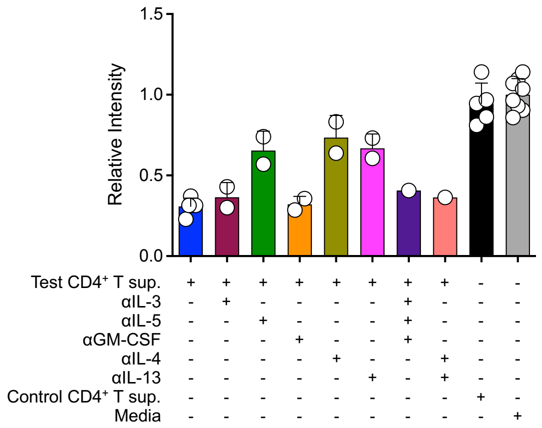

Figure S3. Impact of cytokine blockade on PyMt cell growth-suppressing effect of TSLP-activated CD4⁺ T cell supernatant.

Quantification of crystal violet-stained 2D PyMt cell culture in TSLP-activated WT $CD4^+$ T cell supernatant (test, n=4), test $CD4^+$ T supernatant + αIL-3 (n=2), test $CD4^+$ T supernatant + αIL-5 (n=2), test $CD4^+$ T supernatant + αGM-CSF (n=2), test $CD4^+$ T supernatant + αIL-4 (n=2), test $CD4^+$ T supernatant + αIL-13 (n=2), test $CD4^+$ T supernatant + αIL-3, αIL-5 and αGM-CSF (n=1), test $CD4^+$ T supernatant + αIL-4 and αIL-13 (n=1), $Tslpr^{KO}$ $CD4^+$ T supernatant (control, n=5), and media alone (n=8). Relative intensity of crystal violet stain in each well is determined using ImageJ. Bar graphs show mean + s.d.

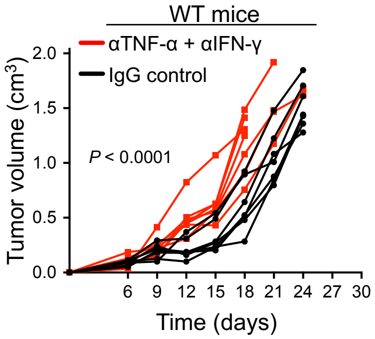

Figure S4. TNF-α and IFN-γ blockade effect on PyMt breast tumor growth in WT mice.

PyMt cell line-derived tumor growth in WT mice treated with anti-TNF-α and anti-IFN-γ blocking antibodies (test, n=7) versus IgG control (control, n=7, $P < 0.0001$, two-way ANOVA).

Supplementary Table

Table S1. Primers used for genotyping mice in the study.

Gene	Forward primer	Reverse primer
$Tslp^{tg}$	TCATCCTGCAAGTACTAGTACGGATGGGGC	TGTTTTGGACTTCTTGTGCCATTTCCTGAG
$PyMt^{tg}$	ATACTGCTGGAAGAAGACGAAATCCTTG	CTCTGTGAGTAGCTCTCATTCTCTGACTC
$TSLPR^{KO}$	AGCGTTGGCTACCCGTGATATTGCTGAAGAG	TCATGAACGACCACTTCCTATGTTGGACACG
